# Construction of a *Bacillus subtilis* and *Escherichia coli* shuttle vector harboring the *fabL* gene as a triclosan selection marker

**DOI:** 10.1016/j.heliyon.2020.e03891

**Published:** 2020-05-13

**Authors:** Elena A. Mordukhova, Jae-Gu Pan

**Affiliations:** a GenoFocus Ltd., 65 Techno 1-ro, Gwanpyeong-dong, Yuseong-gu, Daejeon, 34014, South Korea; b Superbacteria Research Center, Korea Research Institute of Bioscience and Biotechnology (KRIBB), 111 Gwahangno, Yuseong-gu, Daejeon, 34141, South Korea

**Keywords:** Microbiology, Bacterial genetics, Pharmaceutical science, Molecular biology, Triclosan & FabL & shuttle vector & *E.coli* & *B. subtilis*

## Abstract

A new plasmid containing a mutated *fabL* gene from *Bacillus subtilis* as a triclosan selection marker was developed as a useful *B. subtilis*/*E. coli* shuttle vector. The pHT-FabL40 plasmid is stable in both gram-positive and gram-negative hosts with increased plasmid DNA yield in *E. coli*.

Currently, many antibiotic resistance genes have been found but only a part of them are used to select and maintain plasmids in bacterial strains, primarily genes encoding resistance to ampicillin and kanamycin (Hershfield et al., 1974; Sutcliffe, 1978). However, the number of suitable antibiotics for plasmid maintenance may be further limited by the fact that the genes responsible for resistance to chloramphenicol, tetracycline and kanamycin often serve to construct special host strains (Bochner et al., 1980; Yu et al., 2000; Kang et al., 2004). The use of antibiotics for the maintenance of plasmid vectors in *Escherichia coli* seems to be undesirable for many biotechnological goals, such as gene therapy and the production of recombinant proteins for further therapeutic applications (Vandermeulen et al., 2011), and significantly increases the cost of large-scale fermentative production (Kroll et al., 2010).

Recently, many host/plasmid systems without antibiotic resistance genes have been constructed for *E. coli* (Cranenburgh et al., 2001; Hägg et al., 2004) but not for another widely used production strain, gram-positive *B. subtilis*. Triclosan, a nonantibiotic biocidal agent, has been shown to inhibit growth in both gram-negative, *E. coli* (Heath et al., 1998), and gram-positive, *B. subtilis* (Heath et al., 2000), bacteria. A comparison of triclosan-mediated growth showed that growth of *E. coli* DH5α and *B. subtilis* subsp. 168 was inhibited at 0.125 μg/ml and 2 μg/ml triclosan, respectively, versus 3.125 μg/ml ampicillin for *E. coli* DH5α and 6.25 μg/ml chloramphenicol for *B. subtilis* subsp. 168 (Figure 1). These results indicated a higher susceptibility of *E. coli* DH5α and *B. subtilis* subsp. 168 to triclosan compared to ampicillin and chloramphenicol, respectively.

**Figure 1:**
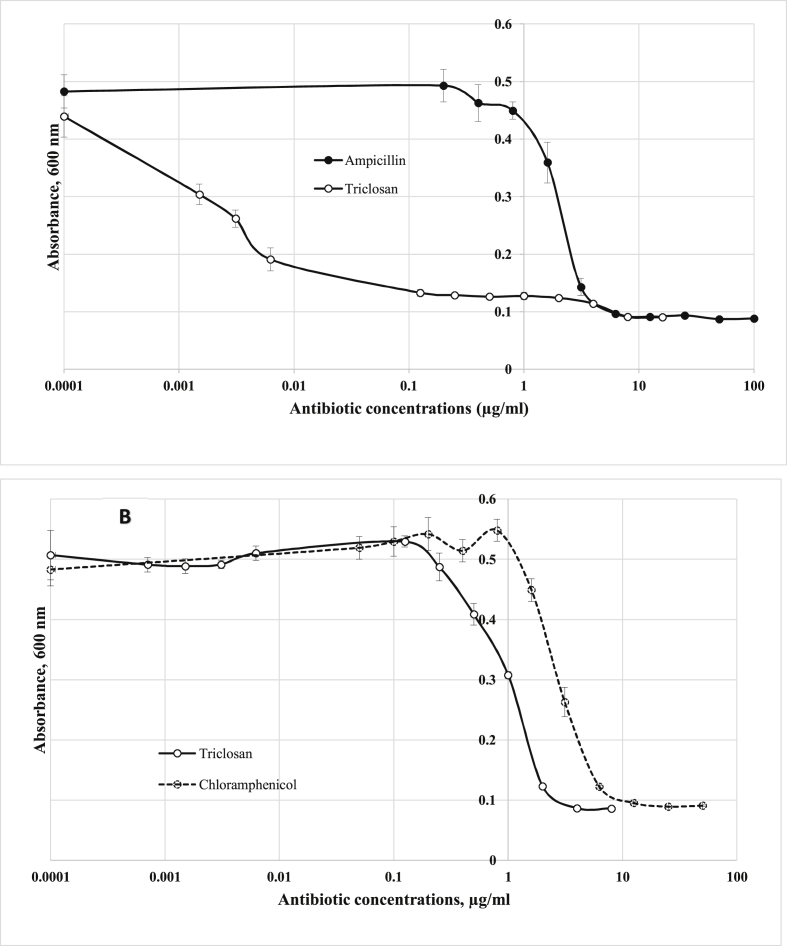
Effect of antibiotics on growth of *E.coli* DH5α (A) and *B.subtilis* subsp. 168 (B). Overnight bacterial cultures were diluted to an optical density (OD_600_) 0.05 in LB medium and grown in the presence of different concentrations of antibiotics in flat-bottom 96-well plates for 12 h at 37 °C in an Infinite 200 Pro microplate reader (Tecan Austria GmbH, Grödig, Austria). Error bars show standard deviations calculated from three independent OD_600_ values for each antibiotic concentration at the end of cultivation.

In *E. coli*, triclosan targets FabI, an enoyl-(ACP) reductase (Heath et al., 1999). The application of the FabI/triclosan selection marker in the pUC19-derived plasmid was first reported by Goh and Good (2008). An improved system with a *fabI* gene harboring a G93V point mutation that confers enhanced resistance towards triclosan was constructed by Jang and Magnuson (2013). Ali and Chew (2015) suggested using the FabV protein, a functional homolog of FabI in *Vibrio cholera*, which confers resistance towards triclosan, for the selection of medium-copy-number plasmids in *E. coli.* This result showed that a foreign protein that is homologous to the *E. coli* protein may be used for plasmid maintenance in *E. coli.* The FabL protein, an enoyl-(ACP) reductase from *B. subtilis,* is responsible for triclosan resistance in the original strain, and the inclusion of the *fabL* gene in high-copy-number plasmids leads to increased triclosan tolerance in *E. coli* (Heath et al., 2000).

In the present study, we developed a *B. subtilis/E. coli* shuttle vector harboring the FabL/triclosan selection marker. This vector was derived from part of the *B. subtilis* pHT01 shuttle expression vector (MoBiTec GmbH, Göttingen, Germany) and contains the *B. subtilis fabL* gene driven by two putative promoters, P2 and P5 (Yamamoto et al., 1999). The fabL gene flanked by a 523 bp 5′ DNA sequence was amplified from *B. subtilis* subsp. 168 genomic DNA using the primers fabL1 (CTGAGCGTGAACAGCTCATTG) and fabL2 (GGACGTCGACTCTAGAGGATCCCGGTCATTAGAATGTGCAGGTG), digested by *EcoRV* and *BamHI* restriction enzymes and inserted into the *BsaBI* and *BamHI* sites of pHT01 by replacing the chloramphenicol resistance gene, *lacI* gene and Pgrac promoter. The ampicillin resistance gene was deleted by the *XhoI/BsaI* digestion of the resulting plasmid, T4 polymerase blunting and self-ligation of the 5.774 kb plasmid fragment. The finally constructed plasmid was named pHT-FabL (Figure 2). In the *E. coli* strain XL-Gold, the *fabL* gene enabled growth in the presence of triclosan at a maximum concentration of 2.5 mg/ml versus 0.25 μg/ml for the plasmid-free strain. After transformation with the pHT-FabL plasmid, *B. subtilis* subsp. 168 grew at triclosan concentrations of 2–2.5 μg/ml, whereas the minimum inhibitory concentration (MIC) value was 1.5 μg/ml for the wild-type strain. To increase the triclosan resistance of the pHT-FabL plasmid, we used random mutagenesis of the FabL protein. A library of randomly mutated *fabL* genes in the pHT-FabL plasmid was generated using the primers fabL3 (CATAAACAATCCTGCATGATAA) and fabL2 and a GeneMorph II EZClone Domain Mutagenesis Kit (Agilent Technologies, Santa Clara, California, USA) according to the manufacturer's instructions. The resulting DNA mixture was transformed into *E. coli* DH5α cells, which were then incubated on solid LB medium supplemented with 2 μg/ml triclosan at 37 °C. The plasmid DNA was purified from the *E. coli* clone cultures and transformed into *B. subtilis* subsp. 168 cells, which were then incubated on solid LB medium containing triclosan (3 μg/ml) at 37 °C.

**Figure 2:**
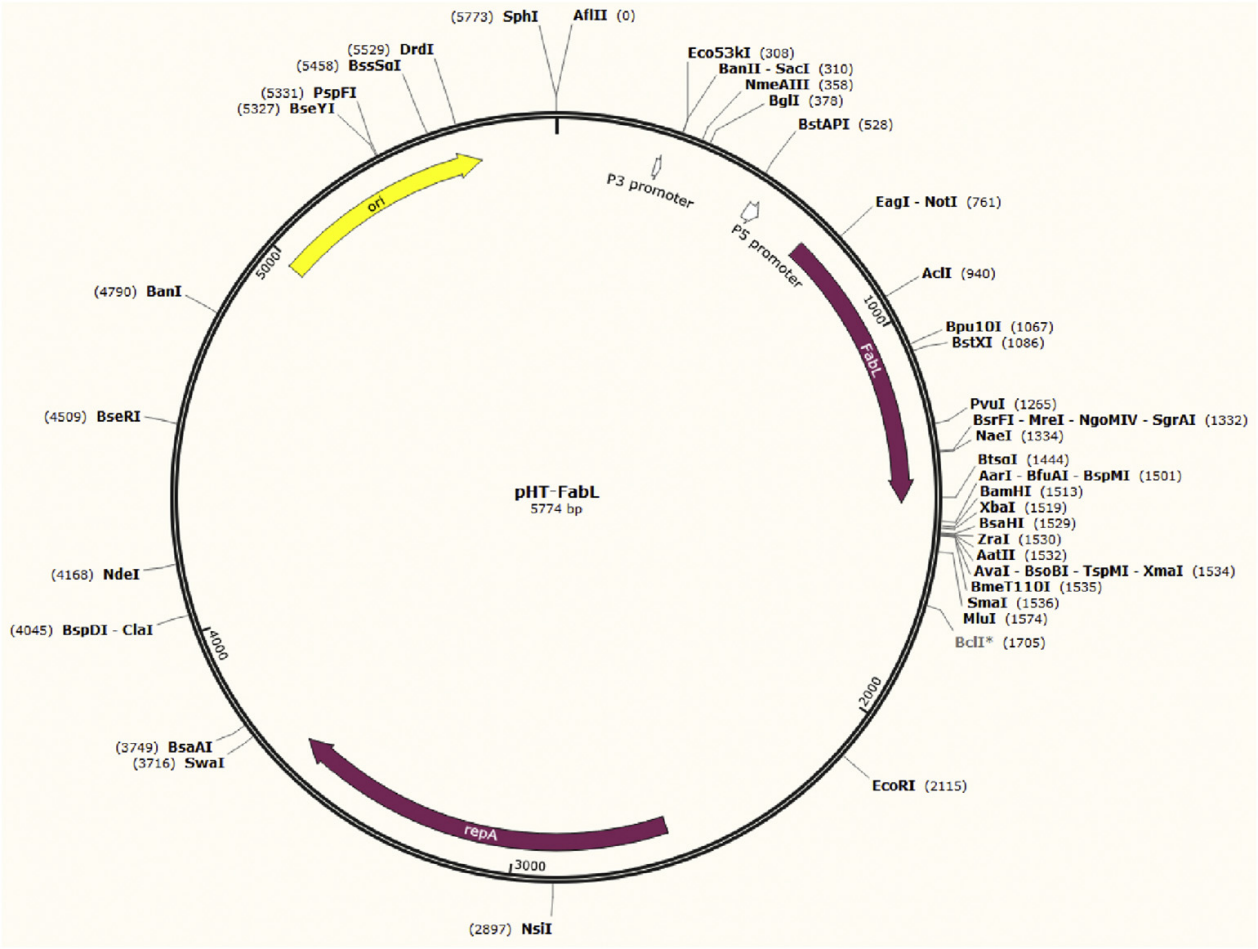
Plasmid map of the pHT-FabL.

To select the pHT-FabL plasmid variant conferring increased triclosan resistance to *B. subtilis* subsp. 168, the growth rates of all plasmid-harboring clones were determined after cultivation in 100 μl of triclosan (3 μg/ml)-containing liquid LB medium in flat-bottom 96-well plates at 37 °C in an Infinite 200 Pro microplate reader (Tecan Austria GmbH, Grödig, Austria; Figure 3). The specific growth rate (μ, h^−1^) was calculated as ln(X/X_0_) using Sigma Plot software, where the initial OD_600_ (X_0_) was 0.15 at the zero time-point, and X was the OD_600_ value 1 h later in an exponentially growing culture. In the presence of triclosan, the *B. subtilis* cells harboring the pHT-FabL40 plasmid grew twice as fast as the cells containing pHT-FabL with the wild-type *fabL* gene (0.179 ± 0.04 vs 0.086 ± 0.02 h^−1^, respectively; Figure 3).

**Figure 3:**
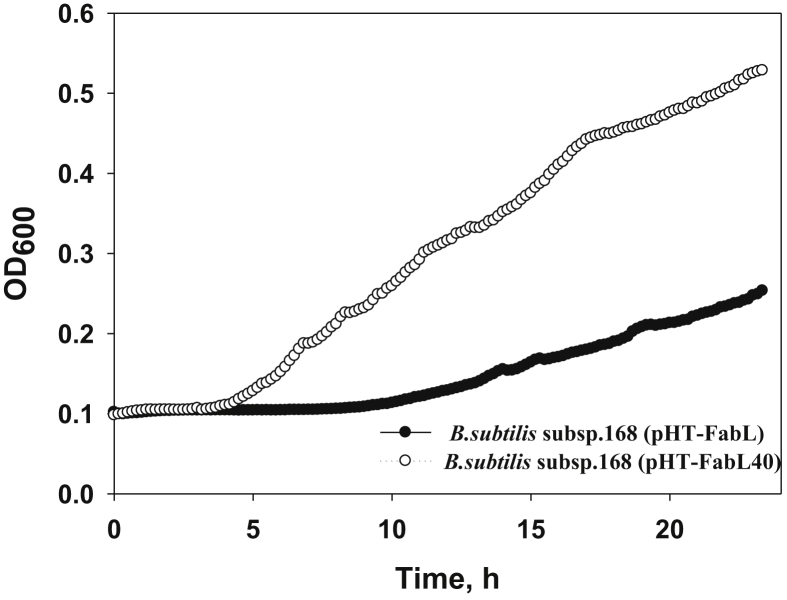
Effect of mutated FabL protein on the growth of *B. subtilis* subsp. 168 in the presence of an increased triclosan concentration (3 μg/ml). All measurements were made in triplicate.

Sequencing analyses revealed the presence of three amino acid substitutions in the *fabL*40 mutant: C_6_F, V_85_I and T_209_M. The cysteine residue at position 6 belongs to the β1-α1 loop of the FabL protein, which has been shown to interact with the pyrophosphate fragment, as well as with additional phosphate in NADP + (Kim et al., 2011). The substitution of threonine for methionine at position 209 may affect the formation of the FabL-NADP-TLC complex (Kim et al., 2011). We suggest that the mutations selected in FabL40 could preserve the catalytic activity of the FabL enzyme in the presence of higher concentrations of triclosan. Confirmation of this opinion is the increased instability index of the FabL40 mutant compared to the wild-type enzyme (33.19 versus 31.10, respectively, https://web.expasy.org/cgi-bin/protparam/protparam). Actually, our proposal is based on the widespread hypothesis that enzymes must trade their stability for higher catalytic activity (Beadle and Shoichet, 2002). We have also detected several nucleotide substitutions upstream of the *fabL*40 mutated gene. One amino acid substitution, Y_2_F, and one substitution of lysine with a termination codon at position 405 were found in the *yfhS* and *yfhQ* (*mutY*) genes located 5’ of the *fabL* gene, respectively. In addition, a G→A point mutation was detected in the P3 putative promoter region of the *fabL* gene. Perhaps these mutations are responsible for higher expression of the *fabL* gene, as was previously shown for the *fabI* in the triclosan-resistant mutant *Staphylococcus aureus* (Grandgirard et al., 2015). The pHT-FabL40 plasmid was retransformed into *E. coli* DH5α, and its stability was studied in comparison to that of the original pHT01 plasmid in gram-negative *E. coli* DH5α and gram-positive *B. subtilis* subsp. 168, as described by Ali et al. (2015) with one modification. The *E. coli* and *B. subtilis* cells harboring the pHT-FabL40 plasmid were cultivated in the presence of triclosan at concentrations of 2 and 3 μg/ml, respectively, which correspond to approximately 7 and 10.5 μM. The cultures of *E. coli* DH5α and *B. subtilis* subsp. 168 retained the pHT-FabL40 and pHT01 plasmids to a similar extent (98 ± 1.36 vs 95 ± 3.54% and 90 ± 1.52 vs 92 ± 2.82%, respectively). The plasmid yield was evaluated according to Ali and Chew (2015). The quantity of the pHT-FabL40 plasmid DNA purified from *E. coli* DH5α was almost three times greater than that of the original pHT01 (3.45 ± 0.08 vs 1.21 ± 0.07 μg/ml, respectively). The results presented here show that the pHT-FabL40 plasmid containing a mutated *fabL* gene as a triclosan selection marker is stable, has an increased plasmid DNA yield, and may be used as a *B. subtilis*/*E. coli* shuttle vector.

## Declarations

### Author contribution statement

Elena A Mordukhova, Jae-Gu Pan: Conceived and designed the experiments; Performed the experiments; Analyzed and interpreted the data; Wrote the paper.

### Funding statement

This work was supported by the 10.13039/501100003715KRIBB Innovation Project.

### Competing interest statement

The authors declare no conflict of interest.

### Additional information

No additional information is available for this paper.
